# Efficacy and safety of radiofrequency ablation versus parathyroidectomy for secondary hyperparathyroidism in dialysis patients: a single-center retrospective study

**DOI:** 10.1038/s41598-022-14623-x

**Published:** 2022-06-18

**Authors:** Mian Ren, Danna Zheng, Juan Wu, Yueming Liu, Chengzhong Peng, Wei Shen, Bo Lin

**Affiliations:** 1 Urology and Nephrology Center, Department of Nephrology, Zhejiang Provincial People’s Hospital, Affiliated People’s Hospital, Hangzhou Medical College, Hangzhou, 310014 Zhejiang China; 2 Department of Ultrasound, Zhejiang Provincial People’s Hospital, Affiliated People’s Hospital, Hangzhou Medical College, Hangzhou, 310014 Zhejiang China

**Keywords:** Developmental biology, Diseases

## Abstract

We compared the efficacy and safety of ultrasound (US)-guided radiofrequency ablation (RFA) and parathyroidectomy (PTX) for the treatment of secondary hyperparathyroidism (SHPT). In this single-center retrospective study, we divided patients into PTX (n = 53) and RFA (n = 47) groups. The primary outcome was the proportion of patients who achieved the target intact parathyroid hormone (iPTH) concentration range (≤ 300 pg/mL). Secondary outcomes were the differences in the changes in iPTH, calcium, and phosphorus levels over time and prognosis. iPTH concentrations of 82.1% and 64.1% in the PTX and RFA groups, respectively, were within the recommended range at the endpoint (P = 0.07). iPTH concentrations in the PTX and RFA groups dropped sharply after treatment (82 ± 163 pg/mL and 280 ± 307 pg/mL, respectively, P < 0.001). There was no difference in the trends of iPTH, calcium, and phosphorus levels between the two groups (P > 0.05). Survival analysis revealed no differences in all-cause mortality and cumulative response rate between the two groups (P = 0.90, P = 0.14, respectively). Notably, the incidence of infection and length of the hospital stay in the RFA group were significantly lower. The preoperative bone-specific alkaline phosphatase concentration was a risk factor for postoperative hypocalcemia. US-guided RFA is minimally invasive and compared to PTX in terms of long-term efficacy and complications in the treatment of severe SHPT in maintenance dialysis patients. It may be used as an alternative technique to PTX; however, further studies are needed.

## Introduction

Persistent calcium–phosphorus–vitamin D metabolism disorders in chronic kidney disease (CKD) stimulate the excessive secretion of parathyroid hormone (PTH), triggering secondary hyperplasia in parathyroid gland tissue and promoting a vicious circle, namely secondary hyperparathyroidism (SHPT)^1^. Fibroblast growth factor 23 (FGF23) resistance, resulting from dysfunction of the FGF23-Klotho axis, also plays an important role. SHPT contributes to bone pain and fracture, accelerates vascular calcification, decreases quality of life, and is one of the main risk factors for death and cardiovascular events in patients with end-stage renal disease (ESRD)^2^.

At present, interventions for SHPT include dietary restrictions, phosphate binders, and active vitamin D analogs such as calcitriol and recent calcimimetics^3,4^. There are certain limitations owing to adverse reactions or drug resistance, and active vitamin D compounds are ineffective for already-formed parathyroid hyperplastic nodules. Therefore, parathyroidectomy (PTX) is the standard therapy for severe drug-invalid SHPT^3,4^. Any form of parathyroidectomy (total or even subtotal) may lead to permanent hypoparathyroidism and adynamic bone disease, requiring long-term calcium and calcitriol supplementation^5^.

However, for patients with multiple complications who cannot tolerate PTX, ultrasound (US) interventions such as microwave ablation (MWA)^6,7^ and radiofrequency ablation (RFA)^8,9^ of the parathyroid glands can be selected. US-guided percutaneous ablation therapy is minimally invasive, reproducible, and has been widely used to treat hyperparathyroidism. Some previous studies showed that MWA or RFA could indeed reduce PTH levels and is effective for SHPT^10–12^, and are not inferior to PTX in terms of safety and efficacy^7,12–15^; however, comparisons of PTX and RFA in terms of hospital stay, economic costs, and especially long-term results are less.

In addition, owing to the sharp drop in PTH after surgery, the rapid intake of calcium by bones leads to “hungry bone syndrome”^16^. Severe hypocalcemia (SH)^17^ can cause convulsions, myocardial dysfunction, seizures, and even sudden death. Therefore, it is important to maintain normal postoperative blood calcium levels. Few studies have shown that preoperative alkaline phosphatase (ALP)^18^ and PTH^19^ levels are independent risk factors for postoperative SH in patients with SHPT^5,20^.

This retrospective study aimed to compare the clinical outcomes, postoperative complications, and long-term prognosis of PTX and RFA in maintenance dialysis patients with SHPT.

## Methods

### Patients

This study was approved by the ethical and scientific review board of the Zhejiang Provincial People’s Hospital. We confirmed that all methods were performed in accordance with the relevant guidelines and regulations of prophylaxis declarations, and the retrospective cohort study included all patients diagnosed with SHPT who underwent PTX or US-guided RFA from June 2014 to December 2020 in our hospital. The inclusion criteria were: (1) 18–85 years old; (2) dialytic vintage ≥ 6 months; (3) preoperative intact PTH (iPTH) concentration > 600 pg/mL; (4) severe SHPT after ineffective medical treatment; (5) at least one hyperplastic parathyroid nodules with diameter ≥ 1 cm found by US examination; and (6) follow-up durations ≥ 3 months. The exclusion criteria were: (1) primary or tertiary hyperparathyroidism; (2) severe cardiopulmonary insufficiency and cannot tolerate treatment; and (3) previous history of PTX or RFA.

### Intervention

All patients underwent routine high-frequency ultrasonography combined with 99mTc-sestamibi SPECT or enhanced CT evaluation for preoperative parathyroid localization. According to the clinical guidelines, combined with the patient's condition, the clinician judged and fully communicated the two methods to the patients. Patients who cannot tolerate general anesthesia or prefer minimally invasive therapy accept RFA. They were assigned into two groups by treatment: PTX group and RFA group.

US-guided RFA was performed by the same doctor who had extensive experience in US intervention and RFA treatment of parathyroid nodules. After disinfection and local anesthesia, 10–30 ml of sterile water was injected around the parathyroid to establish a heat insulation layer to avoid thermal injury to adjacent tissues. Using an iU22 US scanner and a high-frequency linear probe (L12-5) (Philips, The Netherlands), grey-scale imaging was used for guidance, while contrast-enhanced ultrasound (CEUS) with a high-frequency linear probe (L9-3) was used for monitoring. The needle of the 18G radiofrequency electrode (VIVA; STARmed, Goyang, Korea) with a 7 mm active tip was inserted into the target parathyroid nodules to perform thermal ablation. During the procedure, the physician evaluated the patient’s condition and terminated the surgery immediately if hoarseness occurred. During postoperative management, we closely observed whether hematoma or asphyxia occurred. After 4 h, patients gradually resumed eating. We routinely evaluated whether the patients had hoarseness or bucking. If the degree of iPTH decline was unsatisfactory, further examination was needed to determine whether there was a residual or ectopic parathyroid gland.

Total PTX was administered after general anesthesia and routine disinfection. The patient was placed in a supine position. The anterior transverse neck incision was approximately 5 cm along the dermatoglyphic direction. The skin flaps were separated and fixed under the platysma, and the anterior cervical muscles were separated along the white line of the neck. After full exposition of the bilateral thyroid and parathyroid glands, the surgeon carefully dissected and protected the bilateral recurrent laryngeal nerves and then completely removed all visible parathyroid glands. Hemostasis was fully achieved, and the anterior neck incision was sutured layer-by-layer. Parathyroid auto-transplantation (AT) was performed to cut approximately 30–60 mg of parathyroid tissue into 1 × 1 × 1 mm particles and planted on the forearm brachioradialis.

### Clinical data collection

General information on age, sex, dialysis history, clinical symptoms, and treatment procedures was collected. Baseline clinical laboratory variables, such as serum creatinine (Cr), uric acid (UA), albumin (ALB), hemoglobin (Hb), troponin I (TNI), B-type natriuretic peptide (BNP), C-reactive protein (CRP), iPTH, calcium (Ca), and phosphorus (P) levels, and bone metabolism-related indicators such as bone-specific alkaline phosphatase (bALP), beta C-terminal cross-linked telopeptides of type I collagen (β-CTx), N-terminal osteocalcin (N-MID), total type I collagen N-terminal propeptide (tP1NP), and 25-hydroxy vitamin D (25(OH)D), were collected. Clinical data included the number and size of parathyroid nodules, osteoporosis (bone density), carotid arteriosclerosis (carotid artery B-US), hospital stay, and postoperative complications.

### Follow-up and outcomes

Serum iPTH, calcium, and phosphorus concentrations were measured after PTX or RFA at the following time points: 1-month (± 2 weeks), 3-month (± 2 weeks), 6-month (± 1 month), 12-month (± 1 month), and 24-month (± 1 month). All patients were followed until death, kidney transplantation, loss, or the end of the study (June 30, 2021).

The primary outcome was the proportion of patients in the PTX or RFA group with the target range of iPTH concentration during the efficacy assessment phase (follow-up to the endpoint). According to the Kidney Disease Improving Global Outcomes (KDIGO) Guidelines^21^, the target range of iPTH concentration was maintained at approximately 2 to 9 times the upper limit of normal, while goal achievement for iPTH of 124–558 pg/mL, calcium of 2.0–2.5 mmol/L, and phosphorus of 0.97–1.62 mmol/L. According to previous studies and guidelines^19,21,22^, the lowest iPTH level after a successful procedure within 7 days was less than 300 pg/mL. Therefore, our study defined the target range of postoperative iPTH level as less than 300 pg/mL.

The secondary outcomes were the differences in the changes in iPTH, Ca, and P levels over time between the two groups, long-term prognosis (death or recurrence), and occurrence of postoperative adverse events (hoarseness, fever, hematoma, and hypocalcemia). The cumulative response rate was defined as the proportion of patients with iPTH < 558 pg/mL for 3 consecutive months before the end of the study, while the preoperative clinical symptoms were completely relieved. Recurrence was defined as a serum iPTH concentration > 558 pg/mL. Clinically, serum Ca < 2.0 mmol/L was considered to be hypocalcemia, and serum Ca < 1.8 mmol/L was SH, initiating intravenous Ca^5,17^. If serum Ca was between 1.8–2.1 mmol/L in the perioperative period, oral calcium carbonate (1.8–5.4 g/day) and calcitriol (1.0–2.5 μg/day) were given.

### Statistical analysis

All statistical analyses were performed using SPSS version 26.0 for the Mac Sciences version 9.0. The measured data conforming to a normal distribution are displayed as mean ± standard deviation (SD), and the other data are displayed as median and interquartile range. Comparisons between parameters were performed using the independent sample *t* test, Mann–Whitney *U* test, or chi-squared test. A linear mixed model was used to compare serum iPTH, Ca, and P concentrations between the groups during the efficacy assessment phase. Potential predictors of hypocalcemia were analyzed using logistic regression analysis. Survival analyses were calculated by the Kaplan–Meier survival curves. All results were tested using bilateral tests, and significance was set at P < 0.05.

### Statement of ethics

This study protocol was reviewed and approved by the ethical and scientific review board of Zhejiang Provincial People’s Hospital, approval number [2021QT330]. And the study has been granted an exemption from requiring written informed consent.

## Results

### General information

A total of 100 patients underwent therapy between June 2014 and December 2020. There were 53 patients in the PTX group, of which 47 (88.7%) underwent total PTX with AT, 6 (11.3%) underwent total PTX, and 47 patients in the RFA group, of which 26 (55.0%) underwent single-session RFA and 21 (45%) underwent two-session RFA.

The baseline characteristics of the patients are summarized in Table 1. The mean age of the patients was 51 ± 12 years. The average dialytic vintage was 7.8 ± 3.6 years. The median follow-up duration was 30.025 (22.325–38.725) months, and 81% of patients underwent hemodialysis. There were no statistical differences between the two groups in several variables, such as age, sex, dialysis history, follow-up time, renal function, UA, ALB, Hb, CRP, TNI, BNP, iPTH, calcium, phosphorus, and bALP (P > 0.05).

**Table 1:** Patients’ baseline characteristics.

Parameter	PTX Group (n = 53)	RFA Group (n = 47)	P value
Age (years)	50 ± 13	51 ± 12	0.83
Gender, male	66.0%	61.7%	0.65
Dialysis method, hemodialysis	83.0%	78.7%	0.59
Dialytic vintage (years)	7.9 ± 3.8	7.7 ± 3.5	0.82
Follow-up time (months)	31.7 (23.4–42.7)	28.6 (21.3–36.6)	0.09
Nodule numbers	3.9 ± 0.4	3.7 ± 0.6	0.03
Nodule’s maximum diameter (mm)	19.2 ± 5.5	20.3 ± 5.5	0.33
Creatinine (μmol/L)	883.5 ± 251.9	847.3 ± 216.0	0.45
Uric acid (μmol/L)	418.4 ± 118.5	416.7 ± 104.0	0.94
Albumin (g/L)	36.7 ± 4.6	36.40 ± 4.20	0.70
Haemoglobin (g/L)	107.4 ± 18.7	101.0 ± 23.0	0.13
CRP (mg/L)	7.1 ± 10.5	11.1 ± 14.9	0.20
TnI (μg/L)	0.04 ± 0.05	0.04 ± 0.04	0.87
BNP (pg/mL)	158.9 (73.9–422.5)	230.8 (73.7–646.8)	0.32
Baseline iPTH (pg/mL)	1857 ± 812	1747 ± 924	0.53
Calcium (mmol/L)	2.5 ± 0.2	2.5 ± 0.2	0.88
Phosphate (mmol/L)	2.3 ± 0.5	2.2 ± 0.60	0.33
bALP (U/L)	132.3 ± 33.8	146.1 ± 62.7	0.25
β-CTx (pg/mL)	5405.1 ± 951.4	5449.5 ± 843.5	0.82
N-MID (ng/mL)	289.7 ± 391.5	242.3 ± 56.6	0.45
tPINP (ng/mL)	1104.2 ± 296.7	1072.3 ± 260.1	0.60
25(OH)D (ng/mL)	21.8 ± 10.5	21.0 ± 13.4	0.73
Preoperative clinical symptoms, yes	62.3%	53.2%	0.36
Osteoporosis, yes	38.1%	42.3%	0.71
Carotid atherosclerosis, yes	92.3%	76.2%	0.12

*PTX* parathyroidectomy, *RFA* radiofrequency ablation, *CRP* C-reactive protein, *TnI* Troponin I, *BNP* B-type natriuretic peptide, *iPTH* intact parathyroid hormone, *bALP* bone-specific alkaline phosphatase, *β-CTx* beta C-terminal cross-linked telopeptides of type I collagen, *N-MID* N-terminal osteocalcin, *tPINP* total type I collagen N-terminal propeptide.

More parathyroid hyperplasia nodules were detected in the PTX group; there were 3.9 ± 0.4 nodules resected in the PTX group and 3.68 ± 0.63 nodules ablated in the RFA group (P = 0.03). However, there was no difference in the maximum nodule diameter and preoperative clinical symptoms (ostealgia or arthralgia, cutaneous pruritus, skeleton distortion, and calcinosis cutis) between the two groups. The results for bone-derived turnover markers, osteoporosis, and vascular calcification suggested the existence of chronic kidney disease-related mineral and bone disorder (CKD-MBD) in both groups.

### Outcomes

#### Primary outcomes

**Figure 1:**
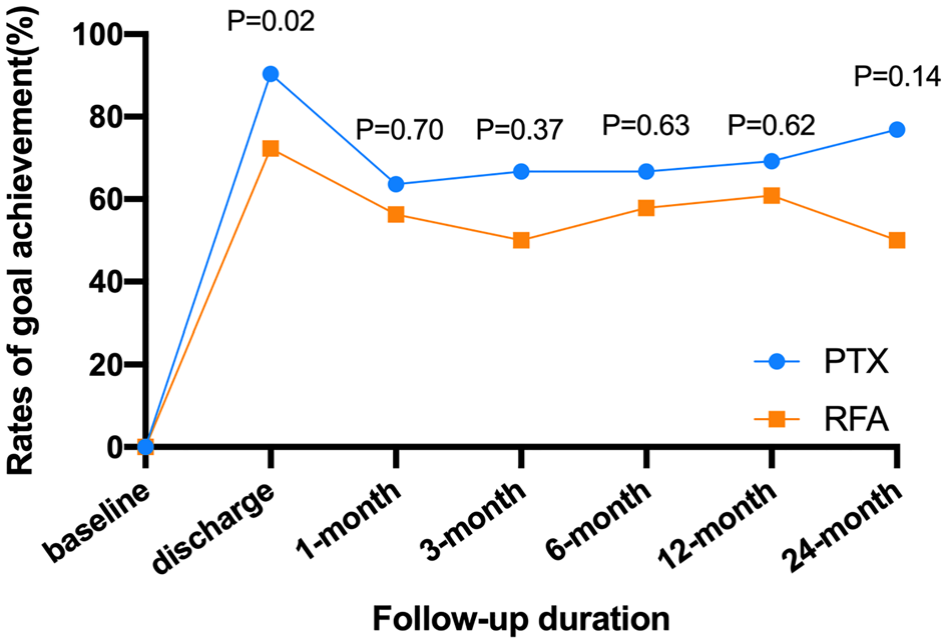
Comparisons of the rates of goal achievement in serum iPTH between the PTX group and the RFA group during the follow-up period.

At the time of discharge, 90.4% and 72.3% of the patients in the PTX and RFA groups, respectively, had iPTH concentrations within the target range (≤ 300 pg/mL) (P = 0.02, Table 2). However, there were no significant differences in the rates of goal achievement for iPTH at any of the follow-up times between the two groups (Fig. 1). In addition, iPTH concentrations of 82.1% and 64.1% in the PTX and RFA groups, respectively, achieved the recommended goal at the study endpoint (P = 0.07). There were significantly more patients with persistently low iPTH concentrations (< 50 pg/mL) in the PTX group than in the RFA group (38.7% vs. 2.6%, P < 0.001), and these patients may have had permanent hypoparathyroidism.

**Table 2:** Proportion of patients achieving and not achieving the target range iPTH concentration by treatment groups.

iPTH level (pg/mL)	PTX Group	RFA Group	P value
**Discharge after treatment**			0.02
≤ 300, No. (%)	47 (90.4)	34 (72.3)	
> 300, No. (%)	5 (9.6)	13 (27.7)	
**Endpoint**			0.07
≤ 300, No. (%)	32 (82.1)	25 (64.1)	
> 300, No. (%)	7 (17.9)	14 (35.9)

#### Secondary outcomes

**Figure 2:**
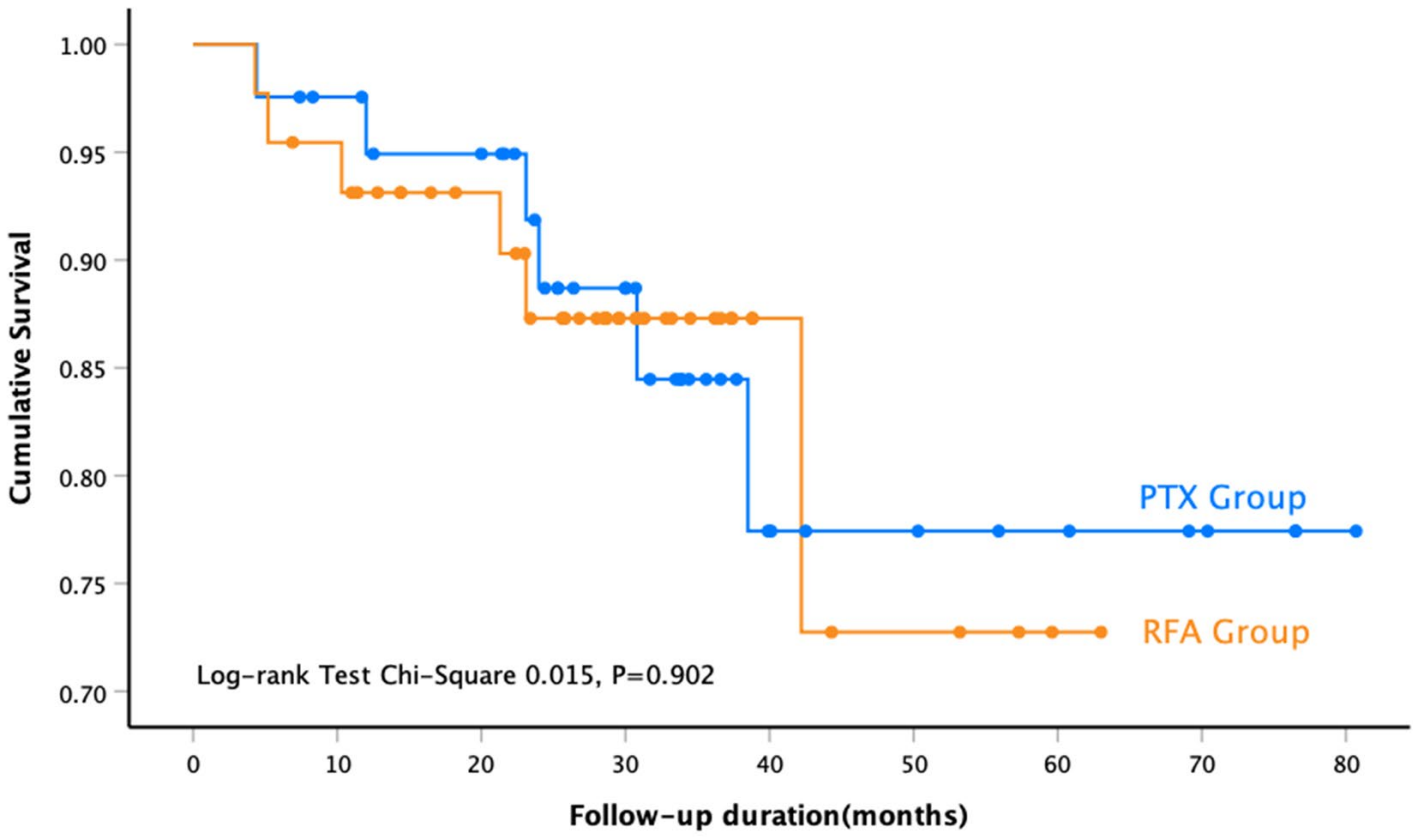
Survival analysis of all-cause mortality after parathyroidectomy or ultrasound-guided radiofrequency ablation.

At the end of our study, a total of 15 patients were lost to follow-up, of which 12 were in the PTX group and three were in the RFA group. Twelve patients died during the follow-up period. The all-cause mortality rates in the PTX and RFA groups were 14.6% (6/41) and 13.6% (6/44), respectively. According to the log-rank test, there was no statistical difference in the long-term survival rates between the two groups (P = 0.90) (Fig. 2). Ten patients relapsed, and the recurrence rates were 9.8% (4/41) and 13.6% (6/44) in the PTX and RFA groups, respectively (P = 0.58). There was no significant difference in the cumulative response rate between the two groups (P = 0.14) (Supplementary Fig. S1).

**Figure 3:**
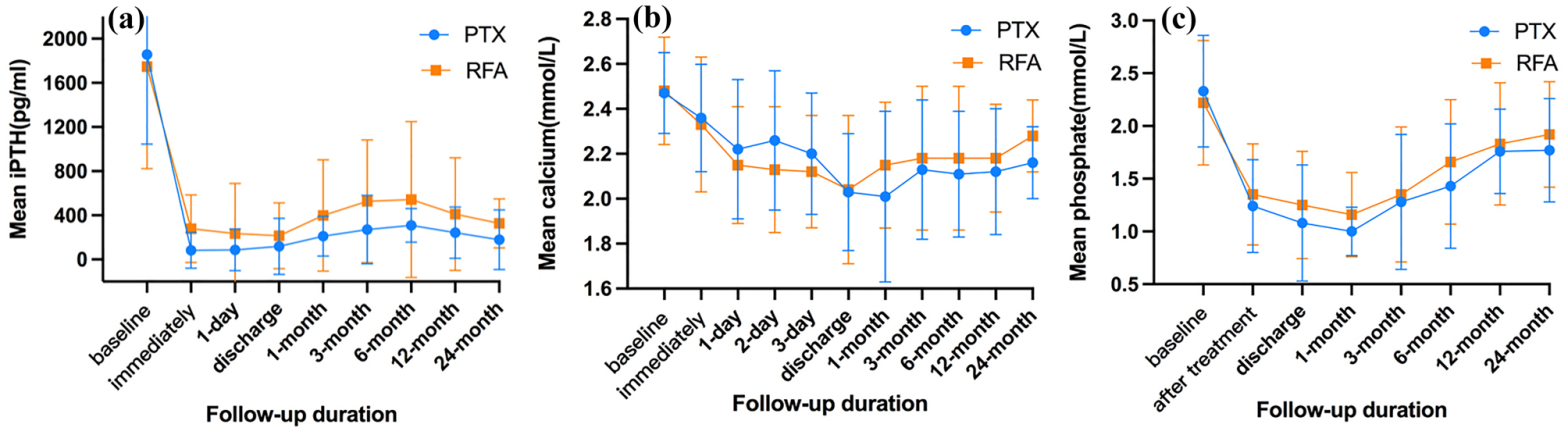
Mean iPTH concentrations (**a**), calcium (**b**), and phosphate (**c**) concentrations in patients treated with parathyroidectomy or ultrasound-guided radiofrequency ablation during the study period. *iPTH* intact parathyroid hormone.

The iPTH concentrations in both groups decreased sharply from baseline after therapy. The mean iPTH concentrations in the PTX and RFA groups immediately after treatment were 82 ± 163 pg/mL and 280 ± 307 pg/mL, respectively (P < 0.001). iPTH levels reached a small peak at 6 months in both groups, and there was no significant difference in the trend over time between the two groups from discharge to 24 months (P = 0.13) (Fig. 3a). The postoperative serum Ca and P concentrations in the two groups were significantly lower than those at the baseline. The average Ca concentration of the RFA group at discharge was the lowest value of 2.0 ± 0.3 mmol/L, while it dropped to the lowest of 2.0 ± 0.4 mmol/L at 1 month in the PTX group. The lowest P concentrations of the PTX and RFA groups were at 1 month: 1.0 ± 0.2 mmol/L and 1.2 ± 0.4 mmol/L, respectively. However, the comparison of Ca and P concentrations at different follow-up times showed no statistical difference between the two groups (P = 0.86 and P = 0.47) (Fig. 3b,c).

### Adverse events and complications

Hoarseness occurred in four and six cases in the PTX and RFA groups, respectively (P = 0.39). Hematoma occurred in three and one patient in the PTX and RFA groups, respectively (P = 0.37). The incidence of hypocalcemia in the RFA group was 55.3%, but there was no significant difference compared with that in the PTX group, which was 43.1% (P = 0.23). SH occurred in 10 patients, including four in the PTX group and six in the RFA group (P = 0.42). The incidence of fever or infection in the PTX group was significantly higher than that in the other groups (P < 0.001). There was a significant difference in postoperative CRP levels between the two groups (P = 0.02) (Table 3).

**Table 3:** Comparison of safe between PTX and RFA group.

Parameters	PTX Group (n = 53)	RFA Group (n = 47)	P value
Total hospital stay (days)	15.5 ± 8.6	11.6 ± 4.5	0.006
Postoperative hospital stay (days)	7.9 ± 5.9	4.5 ± 3.0	0.001
CRP after treatment (mg/L)	34.2 (19.0–88.6)	24.0 (6.5–38.4)	0.02
**Complications**			
Hoarseness, No. (%)	4 (7.5)	6 (12.8)	0.40
Hematoma, No. (%)	3 (5.7)	1 (2.1)	0.37
Fever/infection, No. (%)	21 (39.6)	3 (6.4)	< 0.001
Hypocalcemia, No. (%)	22 (43.1)	26 (55.3)	0.23
Severe hypocalcemia, No. (%)	4 (7.8)	6 (12.8)	0.42

Hypocalcemia < 2.0 mmol/L; Severe hypocalcemia < 1.8 mmol/L.

Compared with the PTX group, the total hospital stays in the RFA group (15.5 ± 8.6 days vs 11.6 ± 4.5 days, P = 0.006) and postoperative hospital stays (7.9 ± 5.9 days vs 4.5 ± 3.0 days, P = 0.001) were significantly less. Furthermore, the RFA group was analyzed using a subgroup analysis. There was no statistical difference between the single-session RFA group and the two-session RFA group in terms of total and postoperative hospitalization time (10.7 ± 4.2 days vs 12.7 ± 4.1 days, P = 0.15; 4.6 ± 3.7 days vs 4.4 ± 1.8 days, P = 0.83). However, in terms of operating expenses, the single-session ablation cost of 1447.70 ± 41.88 $ was less than parathyroidectomy of 1633.85 ± 258.84 $, but the cost of two-session ablation was 2820.25 ± 1.99 $.

### Risk factors of hypocalcemia

**Figure 4:**
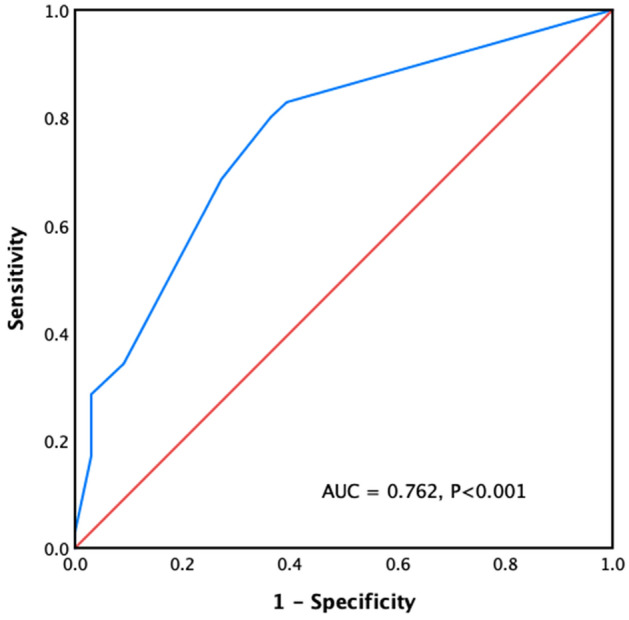
ROC curve of bone-specific alkaline phosphatase (bALP) shows area under the curve of 0.762, sensitivity of 80% and specificity of 63.6% using bALP cutoff point as 115 mmol/L.

Univariate analysis showed that lower age (P = 0.03) and baseline serum Ca concentrations (P = 0.009), higher baseline iPTH levels (P = 0.02), greater reductions in iPTH levels on D1 (P = 0.006), and higher bALP levels (P < 0.001) were associated with a higher risk of hypocalcemia (Table 4). When the cut-off point of bALP level was set at 115 U/L, the area under the ROC curve was 0.762, sensitivity was 80.0%, and specificity was 63.6% (Fig. 4). The results of the binary logistic regression analysis showed an odds ratio (OR) of 1.033 (1.013–1.052); for every increase in bALP level (1U/L), the risk of hypocalcemia increased by 3.3%.

**Table 4:** ROC curves in patients with hypocalcemia.

Variables	Cut-off	AUC	Sensitivity	Specificity	P value
Baseline iPTH (pg/mL)	1210	0.638	0.833	0.36	0.02
Reduction of iPTH (pg/mL)	894.9	0.667	0.979	0.304	0.006
Serum calcium (mmol/L)	2.61	0.654	0.420	0.896	0.009
BALP (U/L)*	115	0.762	0.800	0.636	< 0.001
Age (years)	47.5	0.627	0.700	0.542	0.03

*AUC > 0.7.

## Discussion

In our retrospective study, 100 patients on maintenance dialysis were treated with SHPT. The baseline characteristics of the two groups were similar. Since our hospital had performed PTX as early as 2014 and US-guided RFA began in 2017, the follow-up times were slightly different. In addition, more hyperplastic parathyroid nodules were observed in the PTX group because of their higher visibility.

As a result of decreased kidney function, disordered Ca-P-vitamin D metabolism and increased synthesis and secretion of PTH leads to abnormal bone turnover, mineralization, and vascular or soft tissue calcification, which is clinically diagnosed as CKD-MBD^1,23^. This study showed that approximately 60% of the participants had clinical symptoms such as ostealgia or arthralgia, cutaneous pruritus, skeleton distortion, and calcinosis cutis. Bone-derived turnover markers, such as N-MID, β-CTx, tP1NP, and bALP, were significantly higher than normal. Examination suggested that some patients had osteoporosis and carotid arteriosclerosis. Therefore, the KDIGO guidelines^21^ recommend the treatment of CKD-MBD targeted and maintained at proper ranges of iPTH, Ca, and P concentrations and suggest non-pharmacological therapy for patients with severe hyperparathyroidism.

Most patients in the PTX group underwent total PTX with AT at our hospital. The advantage of this operation is that the function of the transplanted glands can be monitored by measuring iPTH levels in the bilateral forearms^24^. Our study showed that PTX was effective for the treatment of SHPT, which is consistent with the results of previous studies^4,25,26^. Patients who cannot tolerate surgery can be treated with US-guided thermal ablation. Several studies have compared the efficacies of MWA^7,10,26–29^ and PTX in SHPT; however, there are no clear conclusions regarding which method is better. A meta-analysis^30^ revealed that both thermal ablation and PTX were effective treatment options for SHPT, and that thermal ablation increased the risk of recurrence. However, there was no significant difference in the cumulative response rates between the two groups in our study. Moreover, there are currently no definite criteria for choosing the ablation method to treat SHPT. Some studies^6,31^ have compared the effects of RFA and MWA in the treatment of primary hyperparathyroidism and proved that both are safe and effective. Some studies^32–34^ have applied ethanol ablation (EA) to parathyroid adenoma, which can decrease iPTH levels and adenoma volumes; however, it is prone to recurrence and requires multiple injections. There is no consensus regarding the amount of ethanol used or ideal treatment interval^35,36^. RFA appears to be superior to other ablation types in reducing the volume of thyroid nodules, and EA was more effective in the treatment of cyst lesions. Few studies have reported the application of RFA had been applied for SHPT. The results of this study were consistent with those of a previous retrospective cohort study^12^ of 56 patients who underwent US-guided RFA, and showed that iPTH concentrations decreased significantly.

This study compared the clinical efficacy of RFA and PTX and revealed that the iPTH concentrations rapidly decreased, and there were no differences in the iPTH, Ca, and P fluctuation curves between the two groups during the follow-up period. At the study endpoint, the proportions of patients within the target range of iPTH levels in the two groups were similar (82.1% vs. 64.1%, P = 0.07). Moreover, a greater number of patients who underwent PTX had low iPTH levels (< 50 pg/mL). Continuously low PTH levels leads to permanent hypoparathyroidism, which is associated with adynamic bone disease^37^, reduced bone formation, and bone pain or fractures. Thus, it is important to maintain ideal iPTH levels to maintain a normal bone turnover state^38^. As we all known, PTX contributes to reduce the risks of all-cause mortality in CKD patients with SHPT^4,25^, but there are few studies regarding whether RFA is benefit to long-term prognosis. The results of the survival analysis in the present study showed no difference in all-cause mortality between the two groups (P = 0.90). Other researchers^14^ have reported that there was no significant difference in the cumulative death rate between the MWA and PTX groups.

Hypocalcemia is a common complication after invasive treatment of SHPT^5,18^ due to an abrupt decline in iPTH levels. Our results showed that although there was no significant difference in postoperative hypocalcemia between the two groups, the serum Ca levels of the PTX group continued to decrease until 1 month after surgery, which was associated with relatively low iPTH concentrations. bALP^39^ is an extracellular enzyme of osteoblasts, which is a biomarker of osteoblast maturity and bone turnover, and is more accurate and specific than ALP. We found that the postoperative low calcium level in the patients was significantly correlated with a higher baseline bALP level. Previous analysis has indicated that preoperative ALP level is a risk factor for hypocalcemia^5,18,19^. In our study, patients with a baseline bALP concentration > 115 U/L were prone to hypocalcemia, with a sensitivity of 80.0% and a specificity of 63.6%.

The incidence of other complications, including hoarseness and hematoma, was similar between the two groups; however, the incidence of infection in the PTX group was significantly higher (P < 0.001). PTX requires general anesthesia and a longer operation time and is traumatic and invasive^24^. The hospital stay was relatively longer because of prolonged recovery time. In terms of procedural cost, single-session RFA was less effective than PTX. The advantages of US-guided RFA are that it is minimally invasive, fast, and reproducible, and it is no less than PTX in terms of safety and efficacy.

This study had some limitations. First, the number of patients enrolled was relatively small, and larger samples are needed to explore their efficacy and safety. Second, as this was a retrospective study, the results may be biased because some people were lost to follow-up. Prospective studies are needed to verify the therapeutic value of RFA for SHPT and to compare the effects of these two procedures on prognosis using dynamic assessment. Third, because US-guided RFA was performed by the same sonographer, reproducibility may have been affected.

## Conclusion

In summary, US-guided RFA is a promising, safe, effective, and minimally invasive method for severe secondary hyperparathyroidism in maintenance dialysis patients. It may be used as an alternative to PTX, and further prospective studies and randomized controlled trials are necessary to confirm this.

## Supplementary Information

Supplementary Figure S1.

## Data Availability

All data generated or analyzed during this study are included in this article. Further enquiries can be directed to the corresponding author.
