# Supplementary material for: Efficacy and safety of radiofrequency ablation versus parathyroidectomy for secondary hyperparathyroidism in dialysis patients: a single-center retrospective study
Source: Sci Rep. 2022 Jun 18;12:10289. doi: 10.1038/s41598-022-14623-x (PMC9206661; doi:10.1038/s41598-022-14623-x)
Supplement: Supplementary file 1 — Supplementary Figure S1. [file 41598_2022_14623_MOESM1_ESM.pdf]

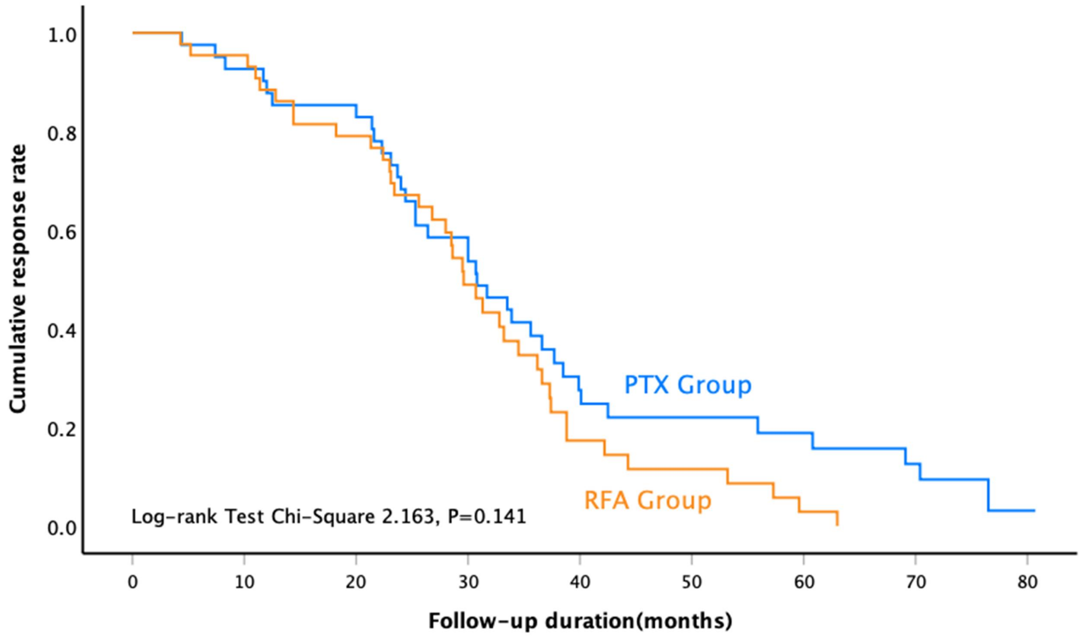

Supplementary Fig. S1 Cumulative response rate of the PTX and RFA group

The cumulative response rate was defined as the proportion of patients with iPTH <558 pg/mL for 3 consecutive months before the end of the study, while the preoperative clinical symptoms were completely relieved.
